# Pulmonary Crohn’s Disease or Crohn’s Disease with Lung Sarcoidosis? A Case Report and Literature Review

**DOI:** 10.3390/healthcare10112267

**Published:** 2022-11-11

**Authors:** Silviu Vlăsceanu, Andrei Bobocea, Cornel Adrian Petreanu, Ioana Anca Bădărău, Horațiu Moldovan, Daniela Gheorghiță, Iulian-Vasile Antoniac, Liliana Mirea, Camelia Cristina Diaconu, Cornel Savu

**Affiliations:** 1 Faculty of Medicine, “Carol Davila” University of Medicine and Pharmacy, 050474 Bucharest, Romania; 2 Department of Thoracic Surgery, “Marius Nasta” National Institute of Pneumology, 050159 Bucharest, Romania; 3 Department of Cardiovascular Surgery, Clinical Emergency Hospital Bucharest, 014461 Bucharest, Romania; 4 Academy of Romanian Scientists, 050045 Bucharest, Romania; 5 Faculty of Materials Science and Engineering, Politehnica University of Bucharest, 060042 Bucharest, Romania; 6 Department of Anesthesiology and Intensive Care, Clinical Emergency Hospital of Bucharest, 014461 Bucharest, Romania; 7 Department of Internal Medicine, Clinical Emergency Hospital Bucharest, 014461 Bucharest, Romania

**Keywords:** Crohn’s pulmonary, sarcoidosis, granulomatous inflammatory disease, extraintestinal manifestation

## Abstract

Background: Crohn’s disease and ulcerative hemorrhagic colitis are forms of granulomatous inflammatory intestinal disease, which usually affects the gastrointestinal tract. There are also reported rare localizations at the skin, kidney, joints, liver and eye level. Pulmonary involvement is relatively rare, and it is most commonly reported in suppuration with bronchiectasis. On the other hand, sarcoidosis is, in principle, a thoracic localization of a granulomatosis disease, although bowel, skin and intestinal disorders are described. There is not a clear line to separate Crohn’s disease from sarcoidosis with, possibly because they are, in fact, considered to have the same inflammatory granulomatosis disease pathology. The diagnoses of the two entities, sarcoidosis and Crohn’s disease, are based on non-pathognomonic, inclusive clinical and paraclinical criteria, without elements of the mutual exclusion of typical locations. Case Report: We present a very rare case of a young male, already diagnosed with small-bowel Crohn’s disease. Granulomatous lung disease with major hemoptysis requires emergency surgery. An intraoperative assessment revealed a necrotic hemorrhagic lesion located in the left lower lobe and a lobectomy was performed. The final pathological report showed the presence of non-caseous granulomatous inflammation, with the identification of specific multinucleated giant cells. Conclusions: The identical diagnostic principles of Crohn’s disease and sarcoidosis, Crohn’s disease as a predecessor to pulmonary lesions, the clinical picture and the necrotico-hemorrhagic appearance of the unilateral pulmonary lesion, which are similar to aggressive necrotico-hemorrhagic or perforating intestinal forms, are arguments in favor of the diagnosis of pulmonary Crohn’s disease and not pulmonary sarcoidosis. At the same time, in general, the two diseases have overlapping elements, suggesting they are, in fact, not the same disease with different facets.

## 1. Introduction

Granulomatous bowel disease and sarcoidosis are non-caseous granulomatous inflammatory diseases of unknown etiology. In addition to their definite localization, both present forms of extensive pulmonary or intestinal manifestations [1,2,3]. The pathogenesis and diagnostic principles, as well as the clinical and paraclinical pictures, overlap and intricate in the extensive forms. As such, it is legitimate to wonder whether it is the same disease or the same pathological manifestation of various disorders in immune or immunoregulatory mechanisms. However, the definition of these diseases was made by the analysis and framing of clinical and paraclinical elements in the absence of a specific diagnostic pathognomonic element, and excluding other granulomatosis that may mimic them, including infectious tuberculosis, histoplasmosis, clostridium difficile or neoplastics. The latter, however, have specific pathognomonic diagnostic elements, including bacteriological or anatomopathological [4,5,6,7].

In 1945, at the American Gastroenterology Association, Watson presented two cases of extensive small-bowel disease with normal radiographs, classifying them as intestinal sarcoidosis. Crohn’s disease itself was refused diagnosis on the principle of a lack of intrathoracic pathological aspects. The board of the association concluded, however, that they were two atypical forms of sarcoidosis. In retrospect, these cases were two typical cases of Crohn’s disease by clinical intestinal pictures [1].

Nowadays, as it was then, the association of Crohn’s disease with respiratory manifestations raises challenges in the correct framing of the disease itself or the association of Crohn’s disease with sarcoidosis, or even two manifestations of the same systemic inflammatory disease.

## 2. Case Report

We present the case of an 18-year-old man with a fever, weight loss and a purulent sputum for 3 weeks, which was the reason for admission to the Pediatrics Department of our hospital. The patient’s past medical history included the diagnosis of small-bowel Crohn’s disease at the age of 14. He underwent immunosuppressive therapy with full remission of the disease. The chest radiography upon admission revealed the presence of left pulmonary infiltrates with mixed air–fluid levels, ranging from a centimeter pattern to a millimeter pattern.

Blood tests identified only mild anemia, with normal coagulation test and biochemistry levels.

**Figure 1:**
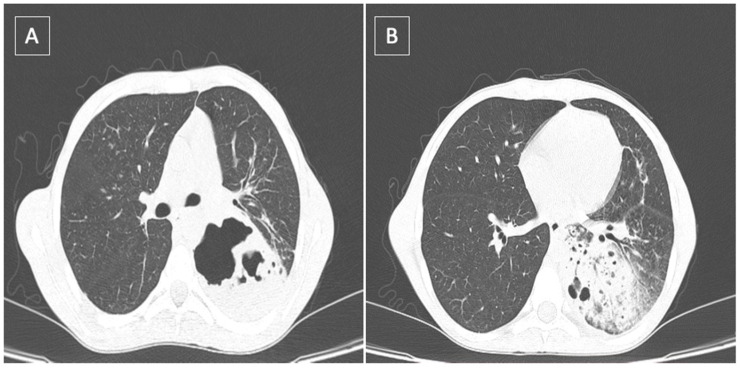
Thoracic CT scan showing: (**A**) lung abscess located in the left lower lobe; (**B**) presence of the left lower consolidation with air bronchogram and perilesional ground glass opacities.

Figure 1 presents a thoracic computed tomography (CT) scan, showing the consolidation areas covering the left lower lobe, cystic bronchiectasis and cavitary pulmonary lesions with mixed gas–fluid levels. A bronchoscopic evaluation found erythematous mucosa of the main bronchial tree, with abundant secretions, specific to chronic bronchiectasis.

The issue was suspected to be pulmonary tuberculosis due to the patient’s history of prolonged immunosuppressive therapy for Crohn’s disease. Tuberculosis treatment was initiated with a quick amendment of the symptoms.

After an episode of massive hemoptysis (400–500 mL of bright-red blood), the patient was transferred to the thoracic surgery intensive care unit. At a second episode of massive hemoptysis (400–500 mL of bright-red blood, within 12 h), an emergency bronchoscopy was performed. Upon completion of the bronchoscopy, we identified the origin of hemoptysis in the lower lobe bronchus, suitable for CT imaging. During investigation, it was difficult to maintain respiration upon the continuous aspiration of blood. Breathing was progressively more difficult due to airway obstruction caused by blood and blood clots.

Single-lung ventilation with a double-lumen endobronchial tube was performed in order to perform surgery and in an attempt to block high-flow hemoptysis. After a left antero–lateral thoracotomy was performed, the lung was inspected. A lung abscess located in the left lower lobe was identified and a lobectomy was performed. The patient recovered uneventfully within one week of discharge. The tuberculosis treatment empirically introduced was ceased.

**Figure 2:**
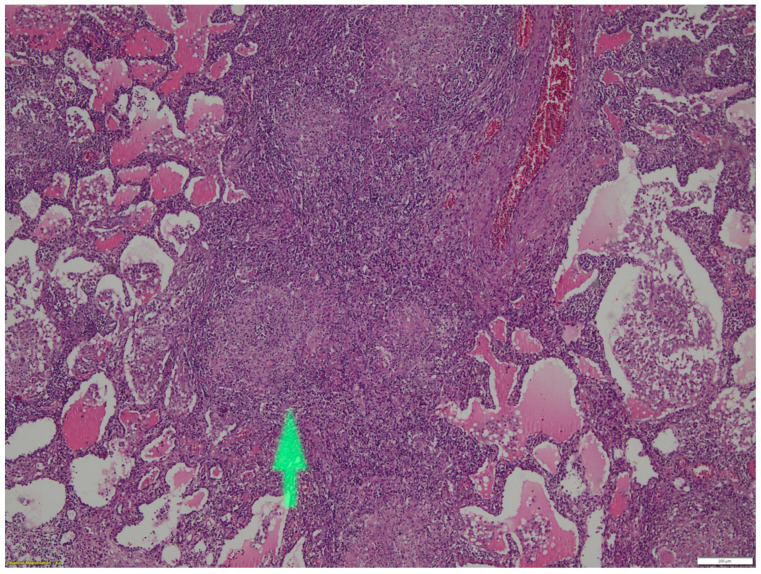
Microscopy imaging, hematoxylin–eosin staining, magnification 4×. The arrow shows the presence of non-caseous granulomatous inflammation with multinucleated giant cells, epithelioid histiocytes, an infiltrate of lymphocytes.

The anatomopathological report is presented in Figure 2, indicating the presence of non-caseous granulomatous inflammation with typical multinucleated giant cells. A granulomatous appearance with epithelioid histiocytes was identified, arranged in nodules, with an infiltrate of lymphocytes and multinucleated giant cells. All data concluded a pulmonary manifestation of Crohn’s disease.

The patient’s follow-up involved regular checkups with clinical examinations, X-rays and thoracic CT scans after 6 months and one year, and no signs of recurrence were identified.

## 3. Discussions

Granulomatous bowel disease, including ulcerative colitis and Crohn’s disease, is an inflammatory granulomatous disease, mainly localized in the intestine, whose diagnostic elements follow three principles: direct and indirect symptoms; histopathological lesions of the intestine; and the exclusion of other possible causes. Crohn’s disease is typically located in the entire intestinal tract, more often the terminal ileum, and it can affect the entire thickness of the intestinal wall. Thus, for diagnosis we have [2]:

- clinical elements, such as digestive symptoms (diarrhea, weight loss, pain, anemia);
- blood biological elements, such as anemia, hypoalbuminemia, malnutrition, inflammatory markers, etc., or coprological analysis;
- imaging and endoscopic targeting of intestinal tract lesions;
- evidence of non-caseous tissue granulomatous lesions;
- exclusion of mainly clostridium-difficile-induced diarrhea.

In the case of the young man presented, the diagnosis included a colonic biopsy, not occasioned by a lower gastrointestinal (GI) endoscopy and the imaging identification of lesions located in the terminal ileum and colon, all in the presence of otherwise unexplained typical symptoms. The diagnosis was indubitable and specific treatment was instituted. Given the presence of moderate symptoms, corticosteroid and mesalazine were administered with significant clinical improvement. After remission at about 6 months, the treatment was limited to mesalazine, and at one year it was stopped, considering the stability and lack of complications.

Extraintestinal manifestations in inflammatory bowel disease are relatively common, occurring in up to 40% of patients [3], but there are authors who claim an even higher incidence [4]. Kuzela et al. [5] identified the occurrence in up to 56–57% of patients, especially Crohn’s disease. Respiratory lesions have been classified into three categories [6]:

(a) Large airway involvement with stenosis, bronchitis, bronchiectasis and pulmonary suppuration;
(b) Parenchymal involvement with lymphocytic alveolitis, parenchymal infiltrates and granulomatous bronchioles;
(c) Reduced lung diffusion capacity in the absence of an imaging counterpart.

This third category is greatly underestimated and underreported, which has led to the inclusion of DLCO (diffusing capacity for carbon monoxide) in routine evaluations of these patients in order to detect subclinical forms and institute inhaled steroid treatment [5,6,7,8,9,10,11,12,13,14,15].

The respiratory symptoms that required hospitalization in the presented case were a fever, decreased weight and a purulent sputum, which initially raised suspicion of pulmonary tuberculosis. Retroactively, the patient confirmed a prolonged but superficial and neglected respiratory symptomatology with a poorly productive cough, asthenia, and a subfebrile temperature. This may suggest an older, undiagnosed lung disease [16,17].

In Crohn’s disease, respiratory impairment is described in the form of bronchiectasis, pneumonia and other suppurative phenomena. The patient was clinico-pathologically classified by this description, a specific aspect being the bilateral affection but with an asymmetry in favor of the left side [18,19,20,21,22,23,24].

Turner and Warwick [25] were the first to describe an existing link between Crohn’s disease and respiratory symptoms in 1968. Kraft et al. [26] published the first original report in 1976, presenting a batch of six patients with inflammatory bowel disease and a chronic purulent sputum. Numerous other authors have described respiratory impairment in inflammatory bowel diseases, implicitly in Crohn’s disease in over 15 reports with many more patients [3,4,5,6,7,8,9,10,11,12,18,19,23,27,28,29,30,31,32,33].

The hemoptysis was a precipitation and was a novelty. The hemoptysis may have occurred secondary to the rupture of the dilated vessels present in the bronchiectasis-type lesions seen in Crohn’s disease with a pulmonary expression, confirmed using computer tomography and bronchoscopic examination. The computer tomography examination also showed the presence of an area of a necrotic abscess, indicating the presence of ulcerative necrotic lesions that may occur in the intestinal wall with subsequent stenosis or perforation. The unilateral phenomena at the left lung level, with the appearance of areas of necrosis and abscess, led to precipitation involving larger caliber vessels with the appearance of fulminant hemoptysis.

Given the impossibility of identifying the time of onset of the respiratory impairment, we did not exclude the possibility that the immunosuppression of the phenomena triggered at the intestinal level during the initial treatment (corticoid + mesalazine) may have favored the appearance of a more intense rebound phenomena at the pulmonary level.

Crohn’s disease (and, implicitly, inflammatory bowel disease) and sarcoidosis are diseases whose diagnosis is based on clinical and paraclinical pictures in the presence of non-caseous granulomatous lesions and in the exclusion of other pathological entities that may have these diagnostic elements. There are numerous reports of pulmonary involvement in Crohn’s disease, gastrointestinal involvement in pulmonary sarcoidosis and actual associations of the two diseases, which raises the legitimate question of whether they are, in fact, not the same disease with different facets.

The criteria for the identification of sarcoidosis are not specific. They are based on the clinical sense of the practitioner to identify the three major elements of diagnosis: clinical and radiological pictures justifying dyspnea; unexplained dry cough, fever and tissue evidence of non-caseous granulomas; and the exclusion of other intrathoracic diseases with similar signs, either inflammatory (tuberculosis, histoplasmosis) or malignant (lymphomas). It has not been established whether it is necessary to prove one or more tissue locations [34].

There are no pathognomonic diagnostic elements. In fact, the basic element in the diagnosis is the dominant clinical and paraclinical picture under the condition of the exclusion of other causes.

The same embryological origin of the lung with the gastrointestinal tract, the similar immune systems in the bronchial and intestinal mucosa, and the presence of circulating immune complexes and antibodies constitute the common basis of the two diseases, Crohn’s disease and sarcoidosis [34,35].

Possible immunoregulatory defects in the granulomatous processes cited represent the exaggerated activity of killer T lymphocytes and natural-killer T lymphocytes, the excess of helper T lymphocytes in areas of disease activity, the identification of circulating immune complexes and specific antibodies [24].

There is no proof of the etiology of the two diseases, but allergens, such as those that are environmental, infectious or genetic (explained by familial forms) are implicated. The diversity of the reports suggests a complex mechanism with the impairment of contact intestinal phagocytosis processes, triggering granulomatous immune mechanisms. It is obvious that these forms of granulomatous diseases occur in large contact surfaces (intestinal or respiratory) with many specific or non-specific triggers. The cutaneous localization would fit the same principle, while the other forms would simply be an expression of the precipitation of immune mechanisms triggered at a distance (arthritis, uveitis, myocarditis, hepatitis).

An extensive literature review of reports on the associations between intestinal Crohn’s disease and thoracic sarcoidosis included identified extensive or limited studies and numerous isolated clinical case reports [1,36,37] (Table 1).

In the case of an association of these diseases, most authors report intestinal localization as the onset form of non-caseous granulomatous disease, followed by the onset of respiratory manifestations. The latter are established during or upon the initiation of drug treatment and immediately or at a distance from colonic resection in the absence of medication. However, there are also forms of onset in the form of sarcoidosis, in which the classification is made directly in the absence of any suspicion of intestinal damage.

Such reports with sequential sarcoidosis–Crohn’s-associated forms are rare. This is the case of a pneumothorax arising from a peripheral parenchymal necrotic granulomatous lesion, in which simultaneous Crohn’s disease was identified by colonoscopy [38]. Montembault et al. [39] also reported the case of a woman in whom the first episode of Crohn’s disease was accompanied by sarcoidosis. Fellerman et al. [40] presented the case of a 21-year-old man who was diagnosed more than 2 years prior with sarcoidosis with a clinical radiological picture of right-lung involvement, and a biopsy confirmed granulomatous lesions with lymphocytosis in bronchoalveolar lavage. At 2 years of age, the patient presented intestinal phenomena of Crohn’s disease, with digestive stenosis and radiological evolution of the left lung. Identically, Kaur et al. [41] presented the case of a young girl in whom the long-term administration of antibiotics and antifungals for the clinical picture of a cough with a sputum, fever did not bring clinical benefit. Subsequently, following a diagnosis of Crohn’s disease and the administration of corticosteroids, respiratory improvement was achieved.

Jonah N Rubin et al. [42] presented a bowel and neurological impairment in a patient already diagnosed with sarcoidosis, claiming extrapulmonary impairments, but no infallible argument was made that it was not Crohn’s disease with concomitant sarcoidosis.

The time of diagnosis of a respiratory disease is, in most cases, after the diagnosis of Crohn’s disease, at variable intervals of time in the order of months or years. The precipitating phenomenon of respiratory diseases could not be clearly identified. A history of the administration of immunosuppressive drugs, such as mesalazine, anti-TNF alpha or corticosteroids, afterwards, has been identified [37,39,40,45,46,47,48,49,50,51,52,53].

Rkiouaka et al. [46] identified pulmonary interstitial forms secondary to anti-TNF treatment with a spectacular response to corticosteroid therapy. The histopathological diagnosis of lung involvement was not mentioned, but it was clinically perfectly superimposable with sarcoidosis. Additionally, as a paradoxical effect of anti-TNF treatment, the appearance of granulomatous pulmonary lesions was recorded in a patient with Crohn’s disease. The direct link was proven by the disappearance of the sarcoidosis picture when the anti-TNF treatment stopped, which could in fact be a rebound to the immunosuppression-induced inflammatory disease with pulmonary expression [54,55].

This dominant feature suggests a greater local exposure of altered immune mechanisms, such as phagocytosis in the gut or local specificity.

In Crohn’s disease, the same types of respiratory lesions are described as in sarcoidosis: lymphocytic alveolitis and interstitial disease with tracheobronchial or parenchymal granulomatous lesions. Radiologically, the two syndromes can also not be differentiated [24].

The clinical forms identified in the Crohn’s–sarcoidosis sequence did not show any degree of specificity. The only particular element was the sequence of suppurative elements after colonic resection. Camus et al. [29] noted pulmonary involvement after a colectomy for Crohn’s disease in the days to weeks following the surgical procedure.

Radiologically, there was no specificity for sarcoidosis, the identification of diffuse pulmonary infiltrates, bilateral nodular forms, macronodular masses or normal radiological findings [24,37].

The histopathological diagnosis of sarcoidosis reported included parenchymatous, lymph node granulomatous lesions and granulomatous bronchial lesions, namely bronchiolitis. In Crohn’s disease, the same types of respiratory lesions are described as in sarcoidosis: lymphocytic alveolitis and interstitial disease with tracheobronchial or parenchymal granulomatous lesions.

Treatment of the concurrent forms was immunosuppressive to diminish the compromised immune chains. However, in general, the therapeutic tactics of the initial disease were changed by imposing a higher step, such as corticosteroid therapy, pulse therapy and anti-TNF alpha.

In support of the same immune mechanism theory of sarcoidosis and Crohn’s disease, Halling et al. [43] identified in a very large study of over 30,000 patients with inflammatory bowel disease a significant risk of developing over 20 other immune-mediated diseases (episcleritis, cholangiolitis, psoriasis, sarcoidosis, asthma, pyoderma gangrenosum, etc.). Obvious correlations have been found between sclerosing angiocolitis, hepatitis and pyoderma gangrenosum. Sarcoidosis was encountered almost twice as frequently compared to the control population.

Massive hemoptysis in sarcoidosis is rarely reported, requiring surgical lung resection or embolization. There are no reports of intestinal digestive damage in selective cases with hemoptysis presented in the literature. Massive hemoptysis forms were secondary to both parenchymal and intrathoracic lymph node forms, with peri-granulomatous hyper-vascularization being incriminated [56,57,58].

Both sarcoidosis and Crohn’s disease can be associated with erythema nodosum, uveitis and arthritis. In addition, CD4 lymphocytes are abundant in the intestinal mucosa, as in sarcoidosis where they can be a pro-diagnostic element [24]. The same author did not identify similarities in ACE levels, but this does not have diagnostic specificity in sarcoidosis either.

An interesting fact is described by Padilla et al. [59] who mentioned the occurrence of sarcoidosis in a lung transplantation from a donor with the disease. This aspect was observed in 8 patients, which indicates the possibility of a transmissible infectious mechanism within transplantation. For sarcoidosis, Propionibacterium acnes, mycobacteria, mold and certain environmental substances, such as pesticides, were mentioned as possible triggers [59]. This assumption, however, does not apply to other transplant patients who will not develop sarcoidosis unless the infectious element is sensitive to the host body’s complementary immune mechanisms. There is also the possibility that transplant-associated immunosuppressive treatment inhibits the mechanisms generating non-caseous granulomatous lesions.

In support of the similar immune mechanisms involved in the development of the two diseases is N. Chebib et al.’s [60] report of the improvement in both Crohn’s disease and sarcoidosis resistant to standard corticosteroid doses in such a pathological association with the administration of anti-TNF monoclonal antibodies.

Intestinal manifestations in sarcoidosis are reported in any segment and can reach 25% of diagnosed forms. Some studies report the involvement of the stomach, duodenum and ileum, but in the absence of systematic biopsy examinations, the presence of these manifestations in sarcoidosis is definitely underestimated. No chronological sequence or anatomical area specificity (ileal, colonic) was identified [61,62].

The largest study of sarcoidosis associated with Crohn’s disease or inflammatory bowel disease more generally is by Jiang Yi et al. [36], who isolated 3995 cases with inflammatory bowel disease and sarcoidosis. Of these cases, 2500 were Crohn’s disease. The authors found general data with a mean age of 54 years and a female dominance (64.8%), but also specific elements. Thus, in the coexistence of sarcoidosis with inflammatory bowel disease, the need for a colectomy, i.e., the presence of severely perforating, more aggressive forms, was reduced compared to the single forms of inflammatory bowel disease (4.9 vs. 2.4%). This phenomenon was explained by the lack of transmural intestinal damage in sarcoidosis-associated forms [1]. This behavior can be translated by more extensive, widespread and more blunted colonic phenomena without necrotico-perforating elements. Identically, penetrating forms of disease were rarer in pathological coexistence. (1.8% compared to 5.6%), which is possibly due to a local decrease in the intensity of the phenomena, with dissipation of the phenomena.

In contrast, those with coexistence showed a higher frequency of respiratory failure (8.1%) than those with inflammatory bowel disease alone, probably due to the extension of respiratory impairment. The subclinical impairment of respiratory function in Crohn’s disease adds to the proven subclinical effects of sarcoidosis. The study concludes that only a certain phenotype of inflammatory bowel disease is associated with sarcoidosis, with a more benign pattern of evolution.

Maamouri et al. [24] observed a specific correlation of certain genetic mutations with sarcoidosis and Crohn’s disease. The CARD15 mutation is found on chromosome 16, especially with SNPs 8, 12 and 13 in Crohn’s disease. These are not found in sarcoidosis cases, for which the HLA B8 and dr3 halogens have been identified.

Gronhagen et al. [44] identified in a case report a familial cluster of sarcoidosis association with Crohn’s disease with the b8 and dr3 haplotypes.

A study of a cohort of nearly 2000 sarcoidosis patients sought to identify genes already shown to be involved in inflammatory bowel disease. The presence of genes involved in IL23 receptor expression with potential importance in the signaling pathway of immune mechanisms was identified. These data support the involvement of the same mechanisms in these diseases [63].

The coexistence of sarcoidosis and Crohn’s disease as manifestations of the same disease was suggested as early as 1947 by Fries et al. [64], but the link between the two entities could not be established. It has been observed that 10% of those with sarcoidosis have bowel disease, and of those with inflammatory bowel disease, including Crohn’s disease, 40–50% have respiratory symptoms.

The National Heart, Lung and Blood Institute stated in support of this theory in 2004 [1]:

(a) both are non-caseous granulomatous inflammatory processes,
(b) the importance of T-helper type 1 cells in the primary immune response involved in both diseases;
(c) the involvement of environmental and genetic factors.

In support of the common genetic basis are the family reports [1,65,66,67]. Willoughby et al. [1] identified a family cluster in three siblings. Two of them developed sarcoidosis and one developed Crohn’s disease in their youth. At the age of 70, one of those with sarcoidosis developed Crohn’s-disease-like phenomena in the absence of features indicating the recurrence of the initial sarcoidosis.

The separation of the two intestinal or respiratory manifestations of inflammatory granulomatous disease is difficult. Many respiratory phenomena are categorized as sarcoidosis unless the intestinal component is excluded and vice versa.

In this case, the young man’s history of Crohn’s disease, the histopathological identification of non-caseous granulomatous lesions and the brutal necrotic hemorrhagic onset of the lesion may support the diagnosis of Crohn’s disease with extraintestinal manifestation, but this is a purely conventional diagnosis.

## 4. Conclusions

The common embryological origin of the intestinal and bronchial mucosa, the similarities of the immune mechanisms in both systems, the clinical and paraclinical manifestations and the common treatment elements of sarcoidosis and Crohn’s disease form the basis for the assertion that there is, in fact, a single inflammatory disease with different facets and different expressions. In this clinical case, the identical principles of the diagnosis of Crohn’s disease or sarcoidosis, the precedent of Crohn’s disease to the lung lesion, the clinical and paraclinical picture, the necrotic hemorrhagic aspect of the lesions unilaterally, similar to the aggressive intestinal necrotic hemorrhagic or perforating forms, constitute arguments in favor of the diagnosis of pulmonary Crohn’s disease, and not pulmonary sarcoidosis following Crohn’s disease. At the same time, the presented case of pulmonary extension of granulomatous bowel disease is surprising in its clinical intensity, secondary to the local ulcerative necrotic phenomena on one side, which supports the diagnosis of pulmonary Crohn’s disease.

Future genetic and immunological analysis will allow the identification of the stage in the primary immunological cascade affected, with specificities for each manifestation of inflammatory granulomatous disease with specific diagnostic elements.

## Figures and Tables

**Table 1:** Literature reports of association Chron’s disease–sarcoidosis.

Author	No. of Cases	Crohn’s Disease (CD)	Sarcoidosis	Specificity
Smith et al. [38]	1 case of CD (young man)	+	+	Pneumothorax
Montembault et al. [39]	1 case of CD (young woman)	+	+	-
Fellermann et al. [40]	1 case of sarcoidosis (young man)	+	+	-
Kaur et al. [41]	1 case of CD (young woman)	+	+	-
Rubin et al. [42]	1 case of CD	+	+	Neurological signs associated
Camus et al. [29]	1 case of CD	+	+	Linked after colectomy
Halling et al. [43]	29 cases (young men) from 30,000 with IBD	+	+	Double ratio to normal population
Jiang et al. [36]	2500 cases of Crohn’s disease from 3995 with IBD	+	+	Lower risks of penetrating disease
Grönhagen-Riska et al. [44]	Familial aggregation	+	+	All Family
Willoughby et al. [1]	Familial aggregation	+	+	Brothers

Abbreviations: CD, Crohn’s disease; IBD, intestinal bowel disease.

## Data Availability

Any further information concerning the case report is available upon request of the corresponding author.
